# Diagnostic Performance of Second-Generation Fujifilm SILVAMP TB LAM in Nigerian Adults with Presumptive Tuberculosis

**DOI:** 10.3390/jcm14217524

**Published:** 2025-10-23

**Authors:** Nadiia Tytarenko, John S. Bimba, Didac Vulcano, Patricia Comella-del-Barrio, Okoedoh Osazuwa, Jacob Creswell, José Domínguez

**Affiliations:** 1 Institut d’Investigació Germans Trias i Pujol, CIBER Enfermedades Respiratorias (CIBERES), Universitat Autònoma de Barcelona, Carretera del Canyet, Camí de les Escoles s/n, 08916 Badalona, Barcelona, Spain or n.tytarenko@stud.onu.edu.ua (N.T.); dvulcano@igtp.cat (D.V.); patricia.comella@gmail.com (P.C.-d.-B.); 2 Microbiology, Virology and Biotechnology Department, Odesa National I.I. Mechnikov University, 65000 Odesa, Ukraine; 3 Zankli Research Centre and Department of Community Medicine, Bingham University, Karu 961105, Nigeria; bimbajs@yahoo.com (J.S.B.); okoedohosazuwa@gmail.com (O.O.); 4 Innovations & Grants, Stop TB Partnership, 1218 Geneva, Switzerland; jacobc@stoptb.org

**Keywords:** tuberculosis, lipoarabinomannan, LAM, HIV, urine

## Abstract

**Background:** Tuberculosis (TB) remains a leading cause of death worldwide, with major diagnostic challenges in low-resource settings. Urine-based lipoarabinomannan (LAM) assays provide a non-invasive option, particularly for people living with HIV who may struggle to produce sputum. Fujifilm has developed a second-generation SILVAMP TB LAM assay (FujiLAM v.2) to improve diagnostic performance. This study aimed to evaluate the diagnostic accuracy of FujiLAM v.2 among Nigerian adults with presumptive TB and directly compare it with the first-generation assay (FujiLAM v.1) using the same set of urine samples. **Methods:** We conducted a retrospective analysis among Nigerian adults with presumptive TB (*n* = 178). Stored urine samples collected in Abuja were retested with FujiLAM v.2 and compared with results previously obtained using FujiLAM v.1 on the same specimens. Xpert MTB/RIF and culture served as the reference standard. Sensitivity, specificity, agreement with the reference standard (Cohen’s Kappa), and differences (McNemar’s test) were assessed. **Results:** FujiLAM v.2 demonstrated sensitivity of 58.3% and specificity of 97.3%, comparable to FujiLAM v.1 (58.3% and 98.0%, respectively). No significant differences were found between test versions across TB or HIV subgroups (*p* > 0.05). Overall agreement between assays was 97.1% (κ = 0.80). **Conclusions:** FujiLAM v.2 showed diagnostic performance consistent with the first-generation assay, with similar sensitivity and specificity to the earlier version. These findings support its potential as a rapid, non-sputum-based diagnostic tool to complement TB testing in resource-limited settings. Further studies are needed to assess its implementation within TB diagnostic algorithms, including studies with multiple production lots.

## 1. Introduction

In 2023, tuberculosis (TB) likely regained its position as the leading cause of death from a single infectious agent, after being surpassed by coronavirus disease (COVID-19) for three years. TB caused nearly twice as many deaths as HIV/AIDS and continues to affect almost 11 million people annually [1]. According to the WHO Global Tuberculosis Report 2024 [1], Nigeria remains among the ten countries with the highest TB burden globally, contributing approximately 4.6% of global incident cases. Although Nigeria accounted for 9.3% of the global increase in notified TB cases in 2023, the TB incidence remains below the estimated national level, indicating that many people with TB remain undiagnosed or unreported.

Persistent diagnostic challenges, including limited accessibility, quality, and timeliness of testing, remain common in low- and middle-income countries. In Nigeria, these challenges are further compounded by limited laboratory infrastructure in rural areas and delays in sputum transport, which hinder timely confirmation and reporting of cases. Additionally, obtaining sputum samples can be difficult for certain patient groups, such as people living with HIV, children, and those with extrapulmonary disease [2]. To address these limitations, research has increasingly prioritized the development of rapid, non-invasive, point-of-care diagnostic methods that do not rely on sputum samples [3,4].

In this context, urine-based assays could be particularly valuable. They can be performed at peripheral health centers, provide results within an hour, and may facilitate earlier diagnosis and treatment initiation in patients who would otherwise be missed. Evaluating urine-based tests in the Nigerian setting is therefore of high public health relevance, as it could complement existing diagnostic tools and help close persistent detection gaps in resource-limited environments.

Urinary lipoarabinomannan (LAM) antigen detection tests have emerged as potential point-of-care diagnostics for TB. However, current urinary LAM assays demonstrate suboptimal sensitivity and are therefore unsuitable for TB diagnosis across all populations. Sensitivity is higher among people living with HIV, particularly those with lower CD4 cell counts [3]. The Alere Determine TB-LAM Ag test (AlereLAM) is the only commercially available and WHO-recommended urine LAM assay [5]. A complex evaluation including 15 studies [6] reported that AlereLAM has a sensitivity of 42% in people living with HIV who have TB symptoms and 35% in those who were not assessed for TB symptoms. However, sensitivity among people without HIV is substantially lower (approximately 10%) [7]. Consequently, there remains a need for novel LAM tests suitable for use across diverse patient populations.

The Fujifilm SILVAMP TB LAM (FujiLAM) point-of-care assay was developed in 2018 (Fujifilm Tokyo, Japan) and has demonstrated enhanced sensitivity for TB diagnosis in patients living with HIV [8]. Additionally, FujiLAM may also be useful for detecting TB in immunocompetent individuals. Since the development of the first version of the test, a large prospective diagnostic accuracy study led by the Foundation for Innovative Diagnostics (FIND) across four African countries, involving 1575 participants, as well as several other studies, revealed significant lot-to-lot variability in FujiLAM’s diagnostic performance [3,8,9]. These findings prompted Fujifilm to refine its manufacturing processes, leading to the development of a second-generation SILVAMP TB LAM test [10].

We evaluated the performance of the second generation of the Fujifilm SILVAMP TB LAM assay (FujiLAM v.2) in patients with presumptive TB, including both people living with HIV and those without HIV, by comparing the results to those previously obtained with the first version (FujiLAM v.1) of the test in the same individuals. To the best of our knowledge, this is the first study to directly compare both FujiLAM versions (v.1 and v.2) using the same clinical specimens, enabling a precise head-to-head assessment of analytical consistency and diagnostic accuracy.

## 2. Materials and Methods

This retrospective study has been described previously [11], but in brief, it included adults (≥18 years) with presumptive TB attending TB diagnostic clinics at district hospitals in Abuja, Nigeria. Patients were consecutively enrolled upon sample submission, regardless of HIV status. Individuals with prior TB diagnosis or treatment within the past year were excluded.

Written informed consent was obtained, and clinical and demographic data were collected via interview. Participants provided sputum and midstream urine samples on-site. Sputum was tested using Xpert MTB/RIF (Cepheid, Sunnyvale, CA, USA) and cultured in duplicate on Lowenstein-Jensen medium. Sputum samples were processed locally for patient care. Urine samples were collected in sterile containers, kept cold, aliquoted into cryovials, and transported frozen to the Germans Trias i Pujol Research Institute (Badalona, Spain) for FujiLAM testing. All urine samples were collected prior to 2021 and were originally tested with the FujiLAM v.1 assay at that time. For the present study, these stored samples were retested with FujiLAM v.2. Because some stored aliquots were insufficient for retesting, a small number of samples were excluded, and FujiLAM v.1 results were recalculated for comparability.

All FujiLAM v.2 tests were conducted from the same manufacturing lot and performed according to the manufacturer’s instructions. Results were interpreted independently by two blinded investigators, disagreements prompted retesting. HIV status was determined using two rapid antigen tests.

The sensitivity and specificity of the FujiLAM v.2 test were assessed using the combined results of Xpert MTB/RIF and culture as the reference standard for bacteriological confirmation. Invalid results were not included in the calculations. Additionally, the results of FujiLAM v.2 were compared with the results of FujiLAM v.1, performed for these urine samples in 2021 [11].

Statistical analyses were conducted using the chi-squared test or Fisher’s exact test for categorical variables, and Student’s *t*-test for continuous variables with normal distributions. A *p*-value of less than 0.05 was considered statistically significant. Concordance between the FujiLAM v.2 test and the reference standard (Culture+Xpert) was evaluated using Cohen’s Kappa coefficient, with values between 0.61 and 0.80 interpreted as indicating substantial agreement and values between 0.81 and 1.00 considered indicative of almost perfect agreement. Differences in paired binary outcomes between test versions were assessed using McNemar’s test, with exact *p*-values reported for small sample sizes. Analysis was performed using MedCalc statistical software, version 23.3.7 (MedCalc Software Ltd., Ostend, Belgium) and GraphPad Prism, version 10.4.1 (GraphPad Software, Boston, MA, USA).

## 3. Results

A total of 178 adults with presumptive TB were enrolled in this study. Of them, 114 (64.0%) were non-HIV coinfected, and 64 (36.0%) were patients living with HIV. The characteristics of the different study groups are summarized in Table 1.

Of the 178 urine samples included in this comparison, 6 were excluded from the FujiLAM v.1 analysis due to invalid results, and 1 sample was excluded from the FujiLAM v.2 analysis for the same reason. Consequently, 172 participants were included in the analysis for FujiLAM v.1, and 177 participants for FujiLAM v.2.

The sensitivity, specificity, and agreement of both FujiLAM versions were assessed using a combined reference standard of culture and/or Xpert MTB/RIF positivity for bacteriological confirmation. This approach was consistently applied across all analyses to evaluate the diagnostic performance of both generations of the test.

The overall sensitivity and specificity of FujiLAM v.1 were 58.3% (14/24) (95% CI = 37–78) and 98.0% (145/148) (95% CI = 94–100), respectively, with an agreement of 92.4% (159/172) (95% CI = 87–96) (κ = 0.641; SE = 0.091) (Table 2 and Table 3). For FujiLAM v.2, the overall sensitivity and specificity were 58.3% (14/24) (95% CI = 37–78) and 97.3% (149/153) (95% CI = 93–99), respectively, with an agreement of 92.1% (163/177) (95% CI = 87–96) (κ = 0.623; SE = 0.092) with the combined reference standard (Table 2).

As summarized in Table 2, both FujiLAM versions showed similar diagnostic performance across all subgroups, with no significant differences in sensitivity or specificity between test generations in either HIV-positive or HIV-negative participants.

Overall, including only valid test results, 97.1% (167/172) (κ = 0.799; SE = 0.087) of the Fujifilm SILVAMP TB LAM results were consistent in both generations of the test. Among the remaining discordant samples, three samples were correctly identified by FujiLAM v.1 compared to the reference standard but misclassified by FujiLAM v.2; two samples were correctly identified by FujiLAM v.2 but misclassified by FujiLAM v.1.

Additionally, five of the six samples that yielded invalid results with FujiLAM v.1 produced valid and concordant results with the reference standard when tested using FujiLAM v.2. The remaining invalid result from FujiLAM v.1 was also invalid in FujiLAM v.2.

To compare FujiLAM v.1 and v.2 results across subgroups, McNemar’s test was performed (Table 3).

Overall, no statistically significant differences were observed between test versions (χ^2^ = 0.2, *p* > 0.65). Similarly, no significant differences were found when stratified by TB status (TB-positive: χ^2^ = 0, *p* = 1.0; TB-negative: χ^2^ = 0.333, *p* = 0.56) or HIV status (HIV-positive: χ^2^ = 0, *p* = 1.0; HIV-negative: χ^2^ = 3, *p* = 0.25). The 95% confidence intervals for discordant proportions were wide in all groups, reflecting small numbers of discordant pairs.

## 4. Discussion

Our evaluation shows that the second-generation FujiLAM assay generally maintains diagnostic performance comparable to its initial predecessor. We observed an overall sensitivity of 58.3% and specificity of 97.3% (using culture/Xpert as the reference standard), essentially matching FujiLAM v.1. These values align with recent studies; for example, Adzemovic et al. found a sensitivity of 54% and specificity of 95% for FujiLAM v.2 in HIV-positive adults [12]. Meta-analyses of FujiLAM studies using strict microbiological reference standards report sensitivity in HIV-negative adults around 53–70% and specificity around 93–99% [7,13], suggesting our diagnostic performance was within the expected range. Huerga et al. similarly reported approximately 60% sensitivity and 87% specificity for FujiLAM in outpatient HIV-positive Africans [3]. Notably, in that study FujiLAM significantly outperformed the AlereLAM test (60% vs. 40% sensitivity) at equivalent specificity but also identified the lot-to-lot variability with false positive results [3]. Our specificity (97%) was higher than some reports (e.g., Székely et al. reported 85% specificity FujiLAM v.1) [9], possibly because of the sample size, and the fact that all the tests performed in our study were from the same FujiLAM v.2 lot. Generally, high specificity was also observed in meta-analyses [7], consistent with our findings. These data confirm that FujiLAM v.2 yields robust accuracy in adults, comparable to both historical v.1 results and other recent evaluations.

Importantly, FujiLAM v.2 performed similarly in HIV-infected and uninfected adults in this study. In the previous evaluation of FujiLAM v.1, we found FujiLAM sensitivity around 66% in HIV-negative adults and 70% in people living with HIV, with very high specificity in both groups [11]. Likewise, Huerga et al. observed higher FujiLAM sensitivity at lower CD4 counts (69% for CD4 < 200) but still substantial detection (47%) at higher CD4 counts [3]. These findings support the notion that LAM can be detected in urine in adult TB cases regardless of HIV status [7,11].

Our McNemar analysis showed no significant difference in classification between FujiLAM v.1 and v.2 in any subgroup (stratified by HIV and TB status), further indicating that performance is consistent across patient types. In practice, then, FujiLAM v.2 potentially could be applied as a point-of-care test in all adults with suspected TB, not only those who are severely immunocompromised.

Lot-to-lot consistency was a major concern with the original FujiLAM, and confirming its resolution remains critical for the new version. Although our study assessed only a single lot of FujiLAM v.2, ensuring reproducibility across multiple production lots is essential before large-scale implementation. Prior multicenter trials documented wide variability in sensitivity and specificity across lots. For example, Székely et al. reported overall sensitivity around 54% and specificity around 85% (against an extended reference standard), with sensitivity ranging from 26% to 73% between sites [9]. Post hoc analysis identified significant variability in the performance of the six FujiLAM lots used, and the authors concluded the test was too variable for clinical decision-making [9]. Huerga et al. similarly found FujiLAM sensitivity ranging from 48–76% and specificity from 77–98% across lots [3]. FujiLAM v.2 was explicitly redesigned to address this issue. Although our study used only one lot of each generation (thus we could not formally test batch consistency), the nearly identical performance of v.1 and v.2 suggests that the core assay performance remains similar. An early report [10] hints that the updated FujiLAM v.2 may indeed have more stable lot-to-lot performance, but larger evaluations using multiple lots are needed. In summary, while v.2 appears to match v.1 in accuracy, confirmatory studies should verify that batch variability has been resolved.

The clinical utility of FujiLAM stems from its speed and ease of use. FujiLAM is a lateral-flow urine test that requires minimal training and equipment. The workflow is simple, electricity is not required, and yields results in under 1 h, making it feasible at peripheral clinics. It is particularly valuable where sputum collection is difficult or delayed. Importantly, FujiLAM is meant to complement existing diagnostics. WHO guidelines emphasize [5] that all patients who can produce sputum should undergo Xpert MTB/RIF (Ultra) as the initial test. Current LAM-based tests are recommended as adjuncts in people living with HIV who are seriously ill, have advanced HIV disease, or cannot promptly provide sputum [5]. Thus, a positive FujiLAM in such individuals could prompt immediate empiric TB treatment and rapid confirmatory testing, whereas a negative FujiLAM would not rule out TB and would be followed by further testing as indicated. In future diagnostic algorithms, FujiLAM could be combined with clinical screening and rapid tests like Xpert to maximize yield.

While promising, and showing performance approaching the minimum WHO Target Product Profile criteria for non-sputum confirmatory tests (≥65% sensitivity and ≥98% specificity), FujiLAM v.2 has not yet fully achieved these benchmarks [4]. Nevertheless, its high specificity and operational simplicity indicate strong potential as a complementary diagnostic if costs of the test do not constitute a barrier.

Our study has limitations. The sample size was modest, particularly among HIV-positive TB cases, which restricts statistical power and yields wide confidence intervals in subgroup analyses. As a result, small differences between test versions may not reach statistical significance, and estimates should be interpreted cautiously. We tested only a single lot of each assay generation, so we cannot assess lot-to-lot reproducibility. We used culture and Xpert on sputum as the reference standard, which may miss some extrapulmonary or paucibacillary cases; thus, our sensitivity could be underestimated and specificity overestimated if any true TB cases were misclassified. Finally, as in other accuracy studies, our population was clinic-based and may not fully represent community screening contexts. Future work should address these gaps. Multicenter trials enrolling larger adult cohorts (with and without HIV), using multiple lots of FujiLAM v.2, would validate consistency and generalizability. Head-to-head comparisons with AlereLAM in the same population would clarify relative performance. Research should also explore FujiLAM as part of multi-step algorithms and assess outcomes like time-to-treatment and mortality. Technically, further improvements could enhance sensitivity toward WHO target profiles (≥65% sensitivity, ≥98% specificity for a non-sputum confirmatory test) [4].

Although FujiLAM offers operational advantages, it is important to note that it is not yet recognized as an official diagnostic tool for tuberculosis case reporting within national programs. For incorporation into surveillance systems, tests must comply with WHO and national regulatory requirements and demonstrate robust multicenter evidence supporting accuracy and lot-to-lot consistency. Broader validation studies and regulatory approvals will therefore be essential for FujiLAM to be integrated into TB diagnostic algorithms and contribute to national case notifications [4,5].

In summary, in adult TB suspects, FujiLAM v.2 demonstrated sensitivity around 58% and specificity around 97%, comparable to the first-generation test and to recent published data [7,12]. Its similar performance in HIV-positive and HIV-negative patients [11] suggests broad applicability. The assay’s simplicity and rapidity make it attractive for decentralized use, particularly where sputum diagnosis is difficult. Addressing issues (as batch consistency and cost) will be important, but if resolved, FujiLAM v.2 could become a valuable tool in adult TB diagnostic algorithms, helping to close the gap in early case detection.

## 5. Conclusions

In this retrospective evaluation among Nigerian adults with presumptive TB, the second-generation FujiLAM v.2 assay demonstrated diagnostic performance comparable to the first-generation test, with high specificity and moderate sensitivity across both HIV-positive and HIV-negative groups. These findings support its potential as a rapid, non-sputum-based point-of-care tool to complement existing diagnostics in resource-limited settings. Further multicenter studies are needed to confirm reproducibility and facilitate regulatory endorsement.

## Figures and Tables

**Table 1:** Demographic information and clinical criteria of participants with presumptive TB.

Variable	All (*n* = 178)	Individuals without HIV (*n* = 114)	Individuals Living with HIV *(n* = 64)	*p*-Value
**Gender**				0.187
Women	101 (56.7%)	60 (52.6%)	41 (64.1%)	
Men	77 (43.3%)	54 (47.4%)	23 (35.9%)	
**Age in years**				**0.027**
Mean (SD)	37.0 (12.9)	35.4 (13.4) ^a^	39.7 (11.7) ^a^	
**TB medication**				0.368
Yes	9 (5.1%)	4 (3.5%)	5 (7.8%)	
No	169 (94.9%)	110 (96.5%)	59 (92.2%)	

SD: standard deviation. *p*-values were obtained using the chi-squared test (or Fisher’s exact test, as appropriate) for categorical variables and Student’s *t*-test for continuous variables. ^a^ Significant differences (*p* ≤ 0.05) are shown in bold.

**Table 2:** Sensitivity, specificity, and agreement of the FujiLAM assay to the reference standard.

		Sensitivity *(%, n/N, 95% CI)*	Specificity *(%, n/N, 95% CI)*	Agreement *(%, n/N, 95% CI)*	K *(SE)*
**FujiLAM** **v.1**	Overall	58.3%, 14/24, 37–78	98.0%, 145/148, 94–100	92.4%, 159/172, 87–96	0.641 (0.091)
	HIV-negative	52.9%, 9/17, 28–77	99.0%, 96/97, 94–100	92.1%, 105/114, 86–96	0.625 (0.112)
	HIV-positive	71.4%, 5/7, 29–96	96.1%, 49/51, 87–100	93.1%, 54/58, 83–98	0.675 (0.152)
**FujiLAM** **v.2**	Overall	58.3%, 14/24, 37–78	97.3%, 149/153, 93–99	92.1%, 163/177, 87–96	0.623 (0.092)
	HIV-negative	58.8%, 10/17, 33–82	96.9%, 94/97, 91–99	91.2%, 104/114, 85–96	0.617 (0.110)
	HIV-positive	57.1%, 4/7, 18–90	98.2%, 55/56, 90–100	93.7%, 59/63, 85–98	0.633 (0.168)

Invalid results were not included in the analysis.

**Table 3:** Comparison of FujiLAM v.1 and v.2 results using McNemar’s test in overall, TB-positive, TB-negative, and HIV status subgroups.

Comparison	^a^ (pos/neg)	^b^ (neg/pos)	χ^2^	*p*-Values ^c^	95% CI (Discordant Proportion)
Overall (all samples)	2	3	0.2	0.65	(0.065–0.817)
TB-positive samples	1	1	0	1.00	(0.013–0.987)
TB-negative samples	1	2	0.33	0.56	(0.008–0.906)
HIV-positive samples	1	1	0	1.00	(0.013–0.987)
HIV-negative samples	0	3	3	0.25	(0.066–0.653)

^a^ Number of samples positive for FujiLAM v.1 and negative for FujiLAM v.2. ^b^ Number of samples negative for FujiLAM v.1 and positive for FujiLAM v.2. ^c^ A *p*-value of ≤ 0.05 was considered indicative of statistical significance.

## Data Availability

The data presented in this study are available on request from the corresponding author. The data are not publicly available due to privacy reasons.
